# Photoreceptor metabolic reprogramming provides survival advantage in acute stress while causing chronic degeneration

**DOI:** 10.1038/s41598-017-18098-z

**Published:** 2017-12-19

**Authors:** Thomas J. Wubben, Mercy Pawar, Andrew Smith, Kevin Toolan, Heather Hager, Cagri G. Besirli

**Affiliations:** University of Michigan, Kellogg Eye Center, Department of Ophthalmology and Visual Sciences, Ann Arbor, Michigan USA

## Abstract

Photoreceptor death is the root cause of vision loss in many retinal disorders, and there is an unmet need for neuroprotective modalities to improve photoreceptor survival. The biosynthetic requirement of photoreceptors is among the highest in the body, and to meet this demand, photoreceptors maintain their ability to perform aerobic glycolysis. This highly regulated form of glycolysis allows cells to efficiently budget their metabolic needs and may be a critical link between photoreceptor function and survival. Pyruvate kinase muscle isozyme 2 (PKM2) is a key regulator of aerobic glycolysis. In the present study, we characterized the effect of PKM2 deletion on baseline functioning and survival of photoreceptors over time by utilizing a photoreceptor-specific, PKM2 knockout mouse model. We found that upon PKM2 deletion, PKM1 is upregulated in the outer retina and there is increased expression of genes involved in glucose metabolism, which led to chronic degenerative changes in the outer retina of these mice. We also discovered that this metabolic reprogramming provided a survival advantage to photoreceptors in an experimental model of retinal detachment. This study strongly supports the hypothesis that reprogramming metabolism may be a novel therapeutic strategy for photoreceptor neuroprotection during acute stress.

## Introduction

The retina has two circulations-retinal, for the inner retinal neurons, and choroidal, which through the retinal pigment epithelium (RPE), feeds the outer retina. Inner retinal neurons are exquisitely sensitive to ischemia, and irreversible inner retinal injury occurs after 2–4 hours of central retinal artery occlusion^1^. On the other hand, the choroidal circulation supplies the outer retina where the photoreceptors are located. Photoreceptors are therefore deprived of oxygen and glucose when they separate from the underlying retinal pigment epithelium, which occurs in retinal detachment. Unlike inner retinal neurons, photoreceptors are able to withstand prolonged periods of oxygen and nutrient deprivation, as demonstrated by numerous clinical studies that show visual gain when the retina is re-apposed to RPE surgically within days to weeks after initial retinal detachment symptoms^2–4^. It is hypothesized that this survival may be largely secondary to the specific metabolic demands and metabolic machinery present in the photoreceptors.

Warburg and colleagues first demonstrated that neoplastic cells convert glucose to lactate despite the presence of oxygen, which is known as aerobic glycolysis or the Warburg effect^5^. In these cells, aerobic glycolysis functions to meet the high demand for metabolic energy and biosynthetic intermediates. Interestingly, Warburg noted that the retina was the only non-proliferative tissue that was capable of aerobic glycolysis and further studies showed that this effect is limited to the photoreceptors^5–8^. It has been hypothesized that the constant turnover of photoreceptor outer segments, with its extremely high metabolic requirements, drives aerobic glycolysis in the mammalian retina^9^. This unique glycolytic specialization includes checkpoints that enable cells to adapt their metabolic state to different physiologic contexts by either shunting glucose to energy production through oxidative phosphorylation or to anabolic processes for biosynthesis^10^. This metabolic flexibility may provide a critical link between photoreceptor function and survival.

Pyruvate kinase muscle isozyme 2 (PKM2) is a key regulator of aerobic glycolysis and catalyzes the final rate-limiting step of glycolysis, converting phosphoenolpyruvate (PEP) and adenosine diphosphate (ADP) to pyruvate and adenosine triphosphate (ATP)^11,12^. Pyruvate can subsequently be reduced to lactate or enter the tricarboxylic acid cycle for high ATP output. PKM2 exists in different enzymatic states. As a tetramer, it displays high catalytic activity and is associated with ATP synthesis and glucose catabolism. The dimeric form has low catalytic activity and is associated with anabolic metabolism and the shuttling of metabolic intermediates to biosynthetic pathways, such as the pentose phosphate pathway that produces nicotinamide adenine dinucleotide phosphate (NADPH) to suppress reactive oxygen species. Nutrient deprivation and allosteric regulators such as serine and fructose-1,6-bisphosphate favor the tetrameric form. Nutrient availability, presence of growth factors, and post-translational modifications, such as phosphorylation of PKM2, favor the nontetrameric, lower activity form^10^.

In accordance with aerobic glycolysis being limited to the photoreceptors in the retina, PKM2 has been shown to localize to photoreceptors^7,13–15^. Likewise, photoreceptors express lactate dehydrogenase A (LDHA), which preferentially converts pyruvate to lactate, a critical step in aerobic glycolysis^14,15^. Recently, genetic perturbation of PKM2 in rod photoreceptors has been shown to decrease the length of outer segments possibly due to a reduced supply of building blocks supplied via aerobic glycolysis, and fibroblast growth factor (FGF) signaling may regulate aerobic glycolysis in the retina via PKM2 tyrosine phosphorylation (Y105)^15^. Additionally, Rajala *et al*. showed that PKM2 tyrosine phosphorylation (Y105) is regulated by both light and phosphoinositide 3-kinase (PI3K)^13^ and this phosphorylation decreases the catalytic activity of PKM2^13^. Yet, the role of this highly regulated protein in baseline photoreceptor survival *in vivo*, as well as during periods of outer retinal stress, remains unknown.

To this end, we characterized the effect of PKM2 deletion on baseline functioning and survival of photoreceptors over time by utilizing a rod photoreceptor-specific, *Pkm2* knockout mouse model. We found that there is a small but significant degeneration of the outer retina in these mice over time. Additionally, in this rod photoreceptor-specific, *Pkm2* knockout mouse model, PKM1 is up-regulated in the outer retina and there is increased expression of genes involved in glucose metabolism. Interestingly, these alterations in the abundance of PKM1 isoform in the outer retina and the up-regulation of the glucose metabolism machinery provided a survival advantage to photoreceptors in an experimental model of retinal detachment.

## Results

### PKM1 and PKM2 protein levels in photoreceptor-specific, *Pkm2* conditional knockout mice

**Figure 1:**
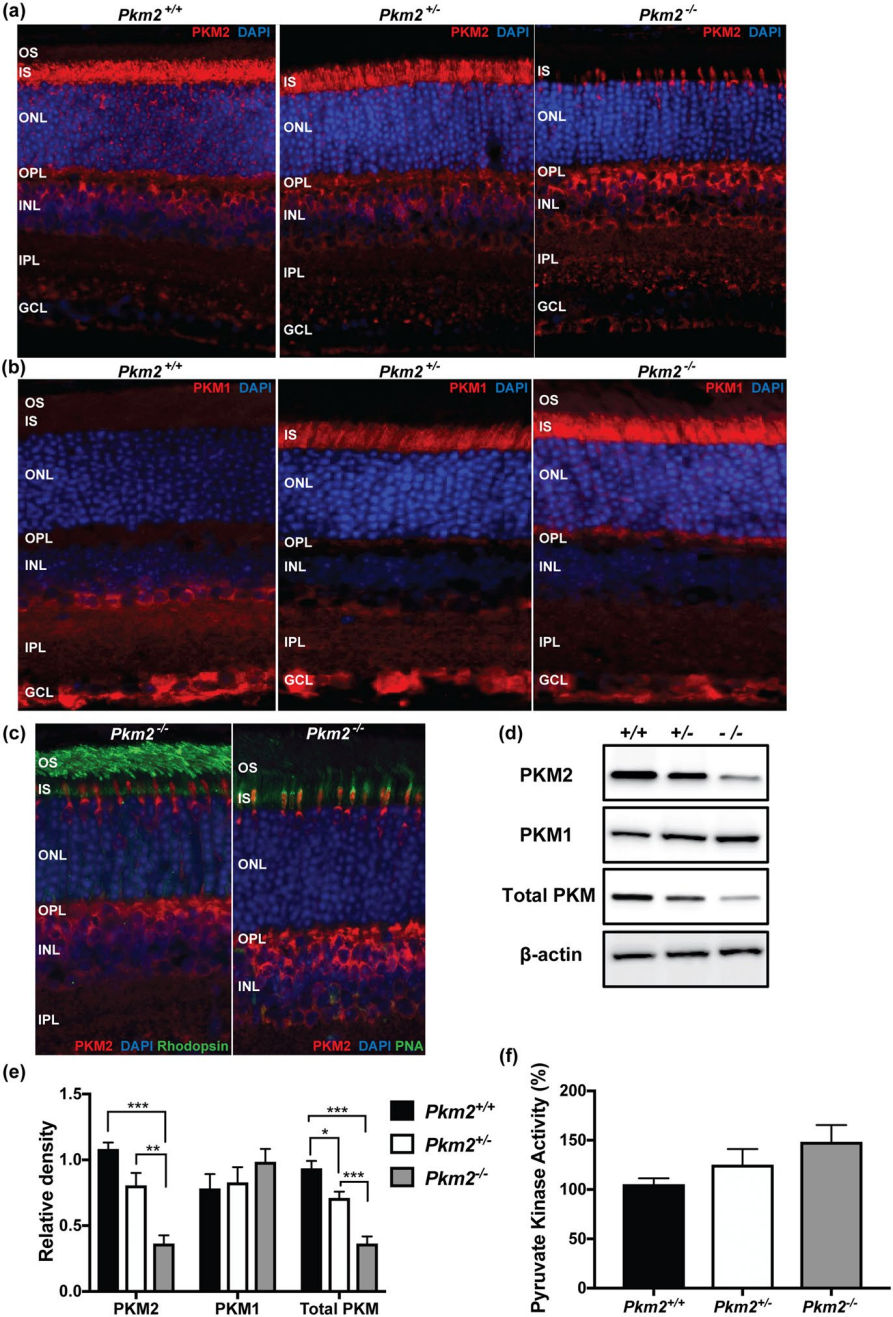
Pyruvate kinase protein levels in photoreceptor-specific, *Pkm2* conditional knockout mice. (**a**) PKM2 immunohistochemistry (*red*) in photoreceptor-specific, *Pkm2* conditional knockout mice shows a decrease in the level of PKM2 in the outer retina with deletion of each *Pkm2* allele from the photoreceptors. (**b**) PKM1 immunohistochemistry (*red)* in these same mice shows increasing levels of PKM1 in the outer retina with deletion of each *Pkm2* allele from the photoreceptors. Nuclei of retinal cells are stained with DAPI (*blue*). OS, outer segments; IS, inner segments; ONL, outer nuclear layer; OPL, outer plexiform layer; INL, inner nuclear layer; IPL, inner plexiform layer; GCL, ganglion cell layer. (**c**) Co-labeling of PKM2 (*red*) and rhodopsin or peanut agglutinin (PNA) (*green*) in photoreceptor-specific, *Pkm2* conditional knockout mice shows the cones retain PKM2 expression in this conditional rod knockout mouse. (**d**) Western blot of PKM2, PKM1, and total PKM showing a decline in the level of PKM2 and total level of PKM in the mouse retina with deletion of each *Pkm2* allele from the photoreceptors. On the other hand, the total level of PKM1 in the mouse retinas increased in the mouse retina with deletion of each *Pkm2* allele from the photoreceptors. β-actin is loading control. (**e**) Quantitative analysis of PKM2, PKM1, and total PKM protein levels in the retina of the photoreceptor-specific, *Pkm2* conditional knockout mice shows a statistically significant decrease in the level of PKM2 and total PKM with deletion of each *Pkm2* allele from the photoreceptors. There is an increasing trend in the level of PKM1 with deletion of each *Pkm2* allele from the photoreceptors. (**f**) Pyruvate kinase activity was measured in mouse retinas using a LDH-coupled enzyme assay. Mean ± SEM; N = 3–5 animals per group; *p < 0.05; **p < 0.01; ***p < 0.005.

Considering the highly regulated nature of PKM2 and its localization to photoreceptors, modulation of PKM2 activity may allow photoreceptors to adapt their metabolic state to specific contexts and ultimately, provide a survival advantage to these cells^7,13–15^. However, the role of PKM2 in the baseline function and survival of photoreceptors as well as during periods of outer retinal stress needs to be further elucidated. To this end, a rod photoreceptor-specific, *Pkm2* conditional knockout mouse model was constructed. In this model, a conditional deletion of *Pkm2* in rods was produced by crossing mice which had the *Pkm2*-specific exon 10 floxed (*Pkm2* ^*fl*^ *°* ^*x/flox*^) with Rho-Cre mice, where Cre-combinase expresses specifically in rod photoreceptors. Mice with both *Pkm2* alleles present in photoreceptors (*Pkm2* ^+/+^), heterozygotes (*Pkm2* ^+/−^), and mice lacking both *Pkm2* alleles in photoreceptors (*Pkm2* ^−/−^) were produced. Similar to previous studies, retinal sections from *Pkm2* ^+/+^ mice stained with antibodies to PKM2 and PKM1 suggest that PKM2 expression is predominantly in the rod inner segments (IS) and outer plexiform layer (OPL) while PKM1 was mainly expressed in the inner plexiform layer (IPL) and ganglion cell layer (GCL) (Fig. 1a,b)^13^. Additionally, as each subsequent *Pkm2* allele was deleted in the *Pkm2* ^+/−^ and *Pkm2* ^−/−^ mice, the amount of PKM2 expression in the rod inner segments decreased significantly (Fig. 1a) with a mild increase in staining in the OPL and inner nuclear layer (INL). This decrease in retinal PKM2 expression was confirmed via western blot analysis (Fig. 1d,e). Double labeling of PKM2 with a rod photoreceptor marker (rhodopsin) and a cone photoreceptor marker (PNA), respectively, confirmed that cone photoreceptors retain PKM2 expression in this conditional rod PKM2 knockout mouse (Fig. 1c). In contrast, as each subsequent *Pkm2* allele was deleted, PKM1 expression in the rod inner segments and outer plexiform layer increased (Fig. 1b). Western blot analysis of PKM1 expression in the retina from each different experimental group of animals showed a similar increasing trend (Fig. 1d,e). This trend in PKM1 expression is similar to what has been previously reported^15,16^. Additionally, total PKM levels were examined via western blot analysis. As each subsequent *Pkm2* allele was deleted in the *Pkm2* ^+/−^ and *Pkm2* ^−/−^ mice, the amount of total PKM declined (Fig. 1d,e). Hence, the upregulation of PKM1 in the outer retina did not equal the decrease in PKM2 levels. However, total pyruvate kinase activity increased with each subsequent *Pkm2* allele deletion (Fig. 1f) in accordance with PKM1 having constitutively high activity.

### Upregulation of genes involved in glucose metabolism in *Pkm2*^−/−^ mice

**Figure 2:**
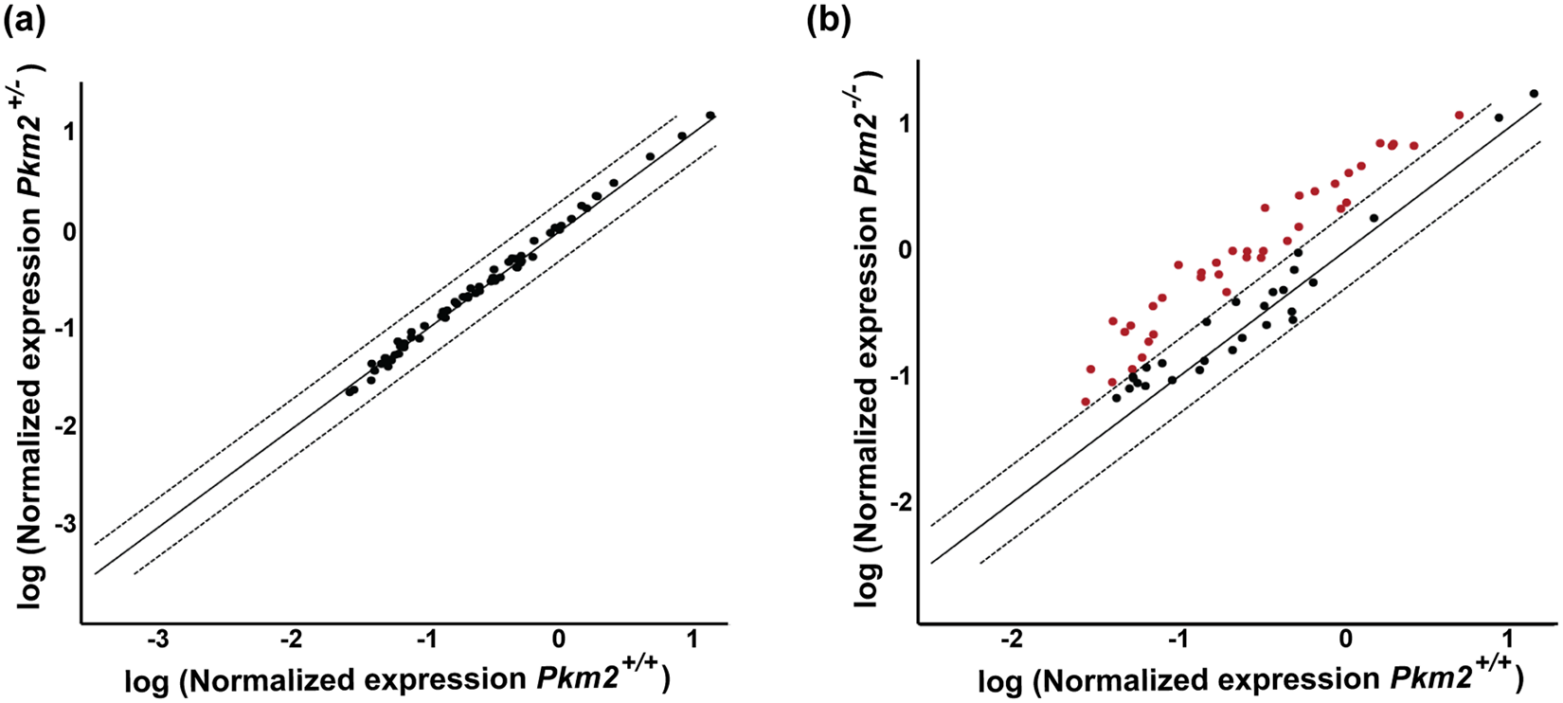
Quantitative PCR analysis of glucose metabolism pathways in retinas from photoreceptor-specific, *Pkm2* conditional knockout mice. (**a**) Scatter plot comparing the normalized expression of genes involved in glucose metabolism between *Pkm2* ^+/−^ mice and *Pkm2* ^+/+^ mice. (**b**) Scatter plot comparing the normalized expression of genes involved in glucose metabolism between *Pkm2* ^−/−^mice and *Pkm2* ^+/+^ mice. The central solid line indicates unchanged gene expression. The dashed outer lines represent the fold change cut-off, which was set to 2. Red solid dots represent those genes that had a fold change greater than 2. Black solid dots represent those genes that did not surpass the fold change cut-off. N = 3–5 animals per group.

As previously discussed, PKM2 is subject to complex regulation and as a result, allows the cell to switch between utilizing glucose and glycolytic intermediates for catabolic versus anabolic metabolism depending on the physiologic state. PKM1, on the other hand, does not have this level of regulation and instead, has constitutively high catalytic activity, which promotes glycolytic flux, ATP synthesis, and catabolic metabolism^10^. As a result, the isoform expression shifts observed in Fig. 1 most likely reduce the photoreceptors ability to dynamically regulate glycolysis in the *Pkm2* ^−/−^ mice. Therefore, to determine if these shifts in PKM1 and PKM2 protein levels alter the expression of genes involved in glucose metabolism, we conducted real-time PCR using the Mouse Glucose Metabolism RT^2^ Profiler™ PCR Array (Qiagen), which examines genes involved in glycolysis, gluconeogenesis, tricarboxylic acid (TCA) cycle, pentose phosphate pathway, glycogen synthesis, glycogen degradation, and regulation of glucose and glycogen metabolism. Using a fold change cut-off of 2, the normalized gene expression patterns of *Pkm2* ^+/−^ and *Pkm2* ^+/+^ mice was very similar (Fig. 2a). However, 37 genes involved in glucose metabolism were found to be upregulated in *Pkm2* ^−/−^ mice as compared to *Pkm2* ^+/+^ mice (Fig. 2b). Notably, the majority of those genes upregulated were involved in glycolysis, the TCA cycle, and the pentose phosphate pathway (Table 1).

**Table 1:** Functional grouping and fold changes of genes upregulated in *Pkm2* ^−/−^ mice.

**Gene**	**Description**	**Fold Change**
**Glucose Metabolism**		
Glycolysis		
*Bpgm*	2,3-bisphosphoglycerate mutase	3.78
*Eno1*	Enolase 1	2.52
*Eno3*	Enolase 3	2.28
*Gpi1*	Glucose phosphate isomerase1	2.69
*Hk2*	Hexokinase 2	6.78
*Pfkl*	Phosphofructokinase, liver	3.99
*Pgk1*	Phosphoglycerate kinase 1	3.70
*Tpi1*	Triosephosphate isomerase 1	3.65
Gluconeogenesis		
*G6pc3*	Glucose 6 phosphatase, catalytic, 3	4.58
*Pck2*	Phosphoenolpyruvate carboxykinase 2	5.00
Regulation of Glucose Metabolism		
*Pdk1*	Pyruvate dehydrogenase kinase, isoenzyme 1	2.37
*Pdk3*	Pyruvate dehydrogenase kinase, isoenzyme 3	4.86
TCA cycle		
*Acly*	ATP citrate lyase	4.04
*Aco1*	Aconitase 1	2.90
*Aco2*	Aconitase 2	2.70
*Idh2*	Isocitrate dehydrogenase 2 (NADP+), mitochondrial	5.24
*Idh3b*	Isocitrate dehydrogenase 3 (NAD+) beta	5.27
*Mdh1*	Malate dehydrogenase 1, NAD (soluble)	3.85
*Mdh2*	Malate dehydrogenase 2, NAD (mitochondrial)	2.41
*Pck2*	Phosphoenolpyruvate carboxykinase 2 (mitochondrial)	5.00
*Pdha1*	Pyruvate dehydrogenase E1 alpha 1	3.90
*Pdhb*	Pyruvate dehydrogenase (lipoamide) beta	2.85
*Sdha*	Succinate dehydrogenase complex, subunit A	2.32
*Sdhb*	Succinate dehydrogenase complex, subunit B	4.81
*Sdhc*	Succinate dehydrogenase complex, subunit C	2.99
*Suclg2*	Succinate-Coenzyme A ligase, GDP-forming, beta subunit	3.90
PPP		
*Rbks*	Ribokinase	3.10
*Rpe*	Ribulose-5-phosphate-3-epimerase	3.52
*Rpia*	Ribose-5-phosphate isomerase A	4.97
*Taldo1*	Transaldolase 1	6.94
*Tkt*	Trasketolase	4.50
**Glycogen Metabolism**		
Glycogen synthesis		
*Gbe1*	Glucan (1,4-alpha-), branching enzyme 1	7.87
*Gys1*	Glycogen synthase 1	2.20
Glycogen degradation		
*Agl*	Amylo-1,6-glucosidase, 4-alpha-glucanotransferase	5.39
*Pygl*	Liver glycogen phosphorylase	4.84
*Pygm*	Muscle glycogen phosphorylase	3.13
Regulation of Glycogen Metabolism		
*Gsk3b*	Glycogen synthase kinase 3 beta	4.60

TCA- tricarboxylic acid; PPP-pentose phosphate pathway.

### Photoreceptor survival in photoreceptor-specific, *Pkm2* conditional knockout mice

**Figure 3:**
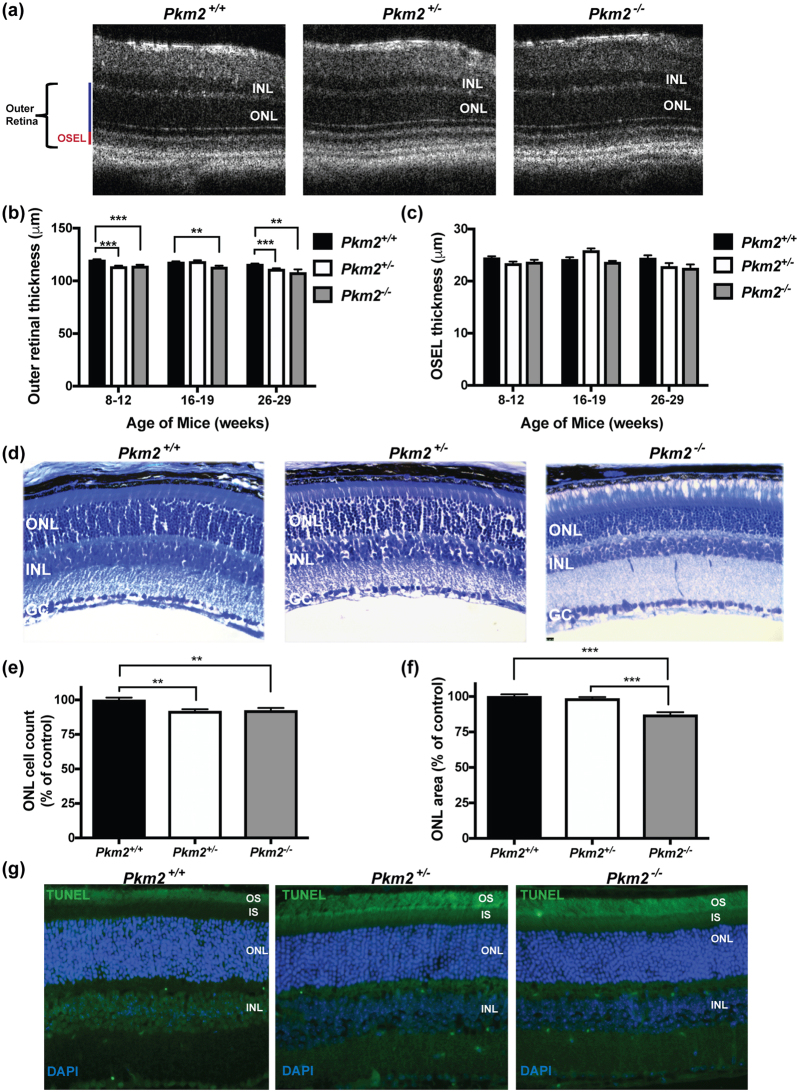
Photoreceptor survival in photoreceptor-specific, *Pkm2* conditional knockout mice. (**a**) Representative OCT images from the photoreceptor-specific, *Pkm2* conditional knockout mice. OSEL, outer segment equivalent length. (**b**) Outer retinal thickness as determined by OCT in the photoreceptor-specific, *Pkm2* conditional knockout mice over time. (**c**) OSEL as determined by OCT in the photoreceptor-specific, *Pkm2* conditional knockout mice over time. n ≥ 7 eyes per group per time point. (**d**) Representative toluidine-blue stained retinal sections from the photoreceptor-specific, *Pkm2* conditional knockout mice. (**e**) Outer nuclear layer (ONL) cell counts normalized to total retinal area per section as a percent of the control (*Pkm2* ^+/+^). (**f**) ONL area normalized to total retinal area per section as a percent of the control. n = 5–9 animals per group; Mean ± SEM; *p < 0.05; **p < 0.01; ***p < 0.005 (**g**) Representative photomicrographs of TUNEL-stained (*green*) retinas from the photoreceptor-specific, *Pkm2* conditional knockout mice. Photoreceptor outer segments showed background staining but there was no true staining in the nuclei of the ONL. Nuclei of retinal cells are stained with DAPI (4′,6-diamidino-2-phenylindole, *blue*). INL, inner nuclear layer; ONL, outer nuclear layer; GC, ganglion cell layer; IS, inner segment; OS, outer segment.

To assess the phenotype resulting from the photoreceptor-specific deletion of *Pkm*2, *in vivo* and *ex vivo* analyses were performed (Fig. 3). Optical coherence tomography (OCT) provides an *in vivo* assessment of retinal structure and thickness (Fig. 3a). A small but significant decrease in outer retinal thickness (outer plexiform layer to retinal pigment epithelium inner surface) was observed in the *Pkm2* ^−/−^ mice as compared to *Pkm2* ^+/+^ mice at all ages (Fig. 3b). The outer retinal thickness of the *Pkm2* ^+/−^ mice varied but a similar small, significant decrease was noted in the 8–12 week and 26–29 week cohorts as compared to the *Pkm2* ^+/+^ mice. Hence, the difference between the *Pkm2* ^+/+^ and *Pkm2* ^+/−^ mice are most likely minor and related to natural variation between animals. None of the changes in the outer segment equivalent length (OSEL, inner segment/outer segment junction to retinal pigment epithelium inner surface)^17,18^ were noted to be statistically significant between the experimental groups (Fig. 3c). In accordance with the *in vivo* observations, *ex vivo* analyses via histology (Fig. 3d) showed statistically significant reductions in the ONL cell counts and area in the *Pkm2* ^−/−^ mouse retinas as compared to those from the *Pkm2* ^+/+^ mice (Fig. 3e,f). These findings were not accompanied by concurrent changes in TUNEL staining, which detect DNA fragmentation in apoptosis and/or necrosis^19^. No significant amounts of TUNEL-positive cells were observed in the ONL under baseline conditions in the different experimental groups (Fig. 3g).

### Retinal function in photoreceptor-specific, *Pkm2* conditional knockout mice

**Figure 4:**
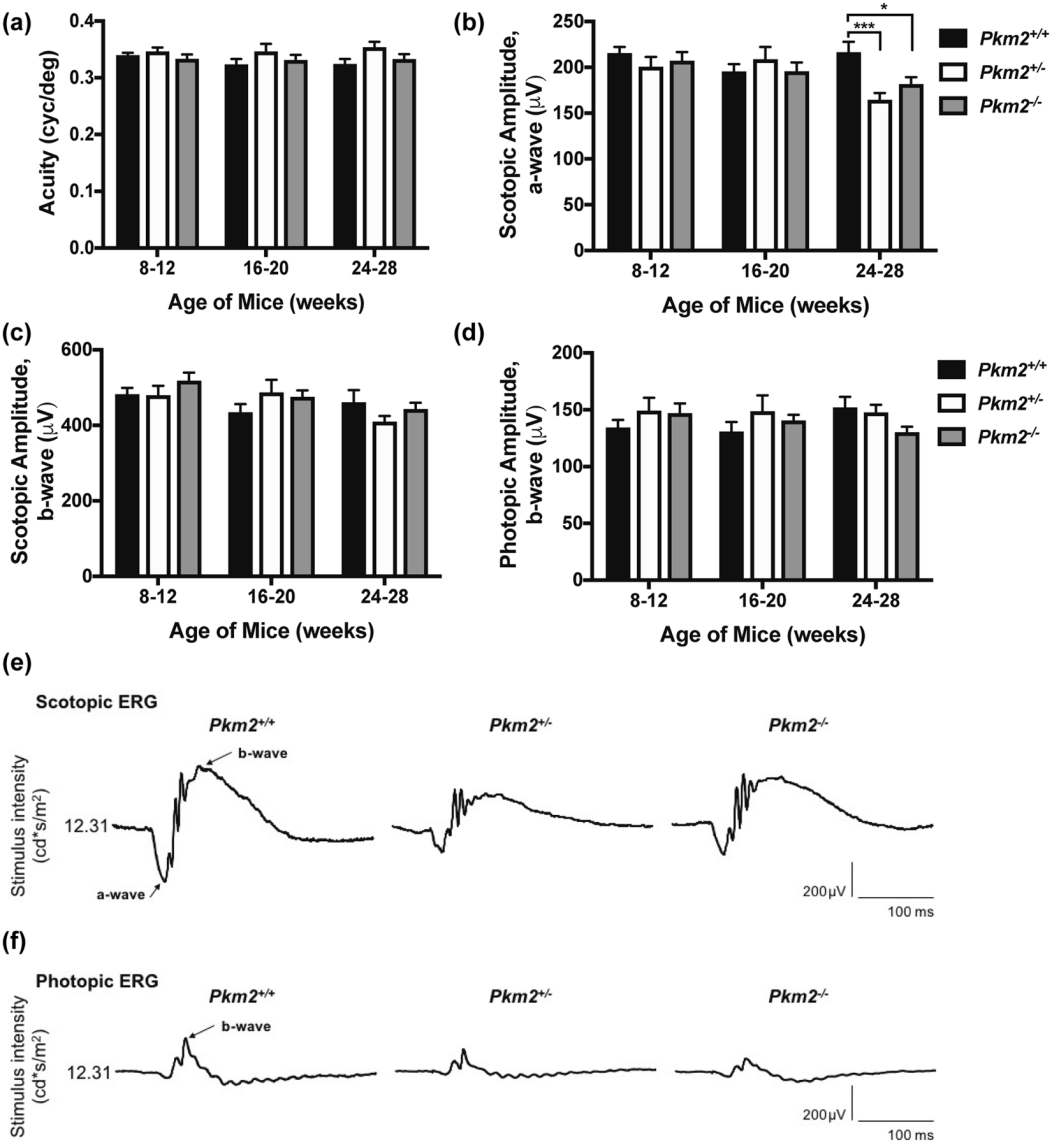
Retinal function in photoreceptor-specific, *Pkm2* conditional knockout mice. (**a**) Visual acuity (cycles/degree) using the optokinetic tracking reflex (Optomotry System, Cerebral Mechanics, Inc., Alberta, Canada) of the photoreceptor-specific, *Pkm2* conditional knockout mice over time. No statistically significant difference between groups or over time. n ≥ 12 eyes per group per time point (**b**) Electroretinography scotopic a-wave amplitudes in the photoreceptor-specific, *Pkm2* conditional knockout mice over time. A flash intensity of 12.31 cd*s/m^2^ was utilized. (**c**) Electroretinography scotopic b-wave amplitudes in the photoreceptor-specific, *Pkm2* conditional knockout mice over time. A flash intensity of 12.31 cd*s/m^2^ was utilized. (**d**) Electroretinography photopic b-wave amplitudes in the photoreceptor-specific, *Pkm2* conditional knockout mice over time. A flash intensity of 12.31 cd*s/m^2^ was utilized. n ≥ 14 eyes per group per time point; Mean ± SEM; *p < 0.05; **p < 0.01; ***p < 0.005. (**e**) Representative scotopic electroretinography (ERG) tracings for the photoreceptor-specific, *Pkm2* conditional knockout mice aged 24–28 weeks. (**f**) Representative photopic ERG tracings for the photoreceptor-specific, *Pkm2* conditional knockout mice aged 24–28 weeks.

Optokinetic tracking and electroretinography were utilized to examine if the anatomical changes observed in Fig. 3 resulted in alterations in visual performance and retinal function. The visual performance of the different experimental groups as assessed by optokinetic tracking (OptoMotry, CerebralMechanics, Inc., Alberta, Canada) did not differ significantly over time (Fig. 4a). Visual function, as evaluated by scotopic electroretinography, showed a small decline in the scotopic a-wave amplitudes of the *Pkm2* ^+/−^ and *Pkm2* ^−/−^ mice over time, and at 24–28 weeks of age, the scotopic a-wave amplitudes of these mice were significantly less than that of the *Pkm2* ^+/+^ mice (Fig. 4b,e). The scotopic and photopic b-wave amplitudes did not differ significantly between the different experimental groups at the ages examined (Fig. 4c–f). Yet, a decline in these amplitudes over time were apparent in the *Pkm2* ^+/−^ and *Pkm2* ^−/−^ mice, similar to that observed in the scotopic a-wave.

### PKM2 tyrosine phosphorylation decreases in experimental retinal detachment model

**Figure 5:**
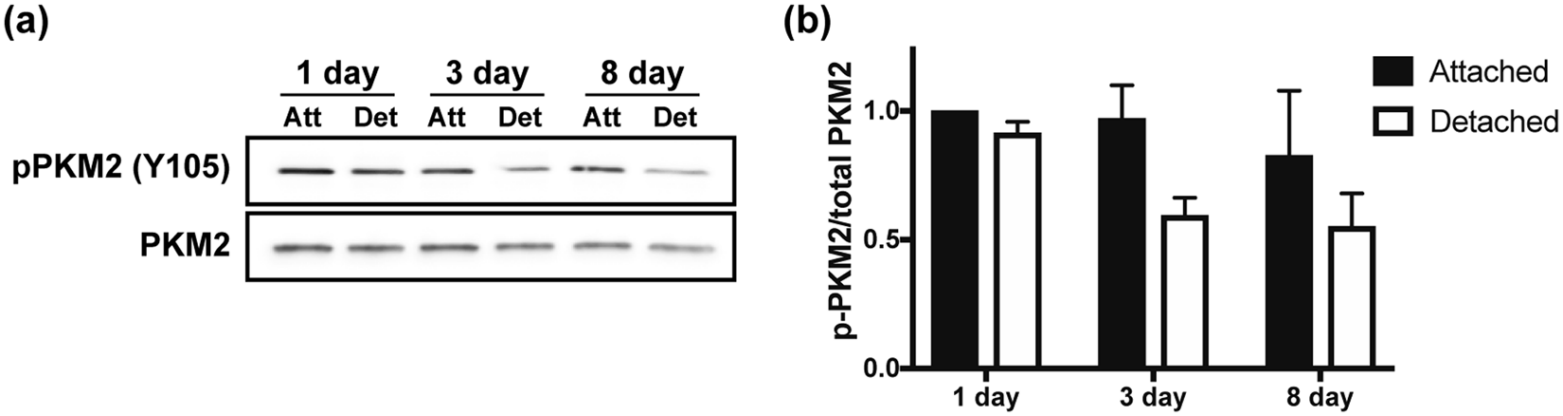
Experimental retinal detachment decreases PKM2 tyrosine phosphorylation. (**a**) Western blot analysis showing decreased levels of PKM2 tyrosine phosphorylation (Y105) as a function of time after experimental retinal detachment in rats. Equal amounts of protein were loaded in each lane. (**b**) Quantitative analysis of phosphorylated PKM2 levels normalized to total PKM2 as a function of time. N = 3 animals per time point; Mean ± SEM; Att-attached retina; Det-detached retina.

PKM2 is subject to complex regulation, which allows the enzyme to switch between a high- and a low-activity state, promoting catabolic or anabolic metabolism, respectively^10,11^. One of the post-translational modifications that alter the activity of PKM2 is the phosphorylation of tyrosine 105 (Y105). Phosphorylation of this amino acid results in decreased catalytic activity of PKM2, possibly by preventing stabilization of the highly active tetramer^11,13^. Conversely, abolishing this phosphorylation site leads to increased glycolytic activity, decreased lactate production, and increased oxygen consumption^20^. Additionally, it has recently been shown that PKM2 tyrosine phosphorylation in the retina is light-dependent and regulated by FGF signaling^13,15^. Therefore, we sought to determine if PKM2 tyrosine phosphorylation (Y105) was regulated under acute outer retinal apoptotic stress^21,22^. Rat retinas were detached and harvested after 1, 3, and 8 days. Western blot analysis showed that experimental retinal detachment leads to a decrease in the levels of PKM2 tyrosine phosphorylation over time (Fig. 5).

### *Pkm2*^−/−^ mice show decreased photoreceptor death in retinal detachment model

**Figure 6:**
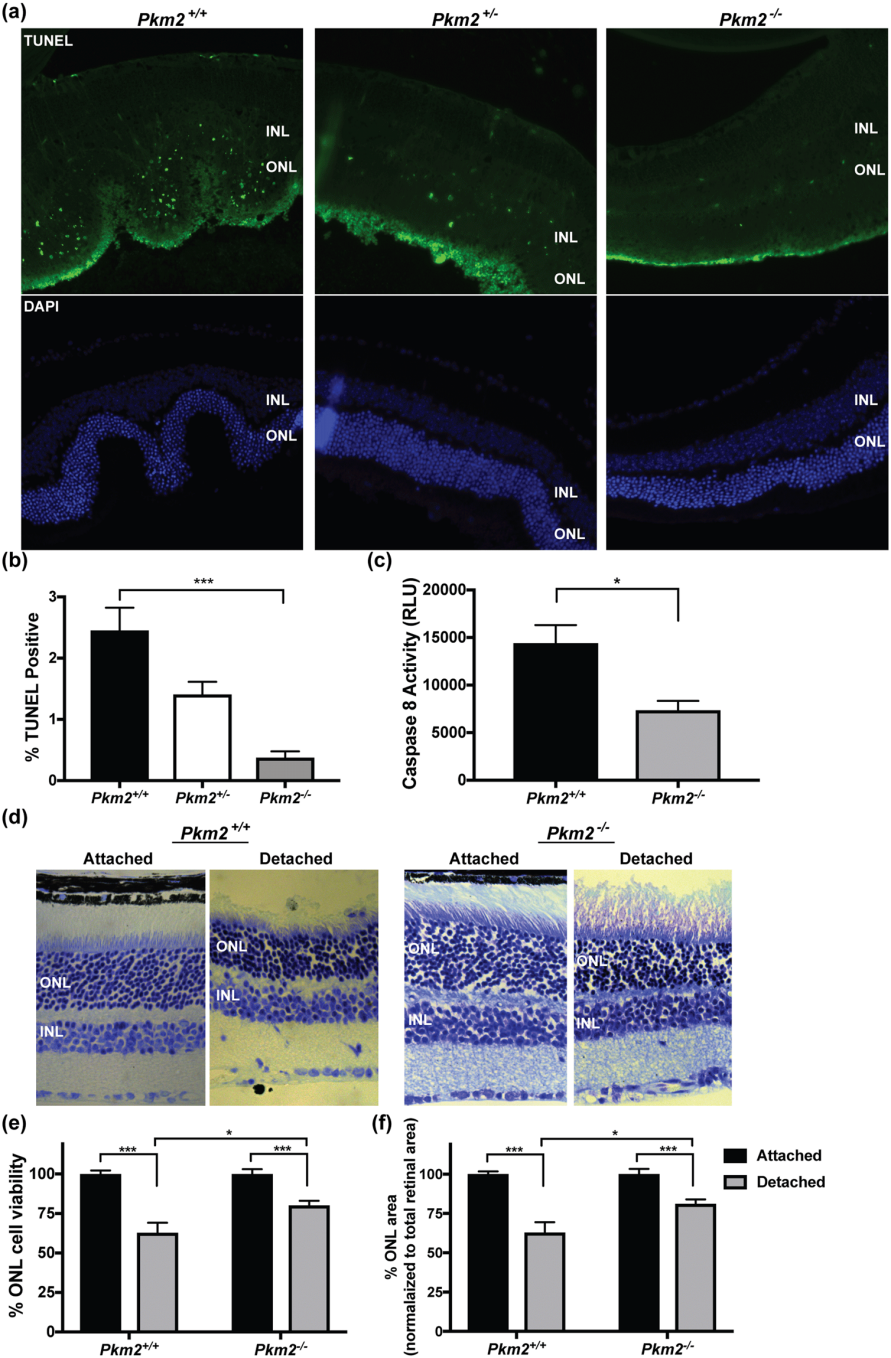
Improved photoreceptor survival after retinal detachment in *Pkm2* ^−/−^ mice. (**a**) Representative photomicrographs of TUNEL-stained photoreceptors (*green*) after 72 hours of retinal detachment. Nuclei of retinal cells are stained with DAPI (*blue*). INL, inner nuclear layer; ONL, outer nuclear layer. (**b**) Quantification of TUNEL-positive cells in the ONL. n ≥ 12 sections per group. (**c**) Caspase 8 activity in detached retinas after 3 days as detected by luminescent assay. N = 3 animals per group; Mean ± SEM; *p < 0.05; ***p < 0.005. (**d**) Retinal sections of attached and detached retina, 2 months after creation of detachment in *Pkm2* ^+/+^ and *Pkm2* ^−/−^ mice, respectively. (**e**) Graph summarizing ONL cell viability between attached and detached retina after 2-month detachment. Cell survival was significantly higher in *Pkm2* ^−/−^ mice as compared to *Pkm2* ^+/+^ mice. (**f**) Graph summarizing ONL area changes between attached and detached retina after 2-month detachment. ONL area was significantly higher in *Pkm2* ^−/−^ mice as compared to *Pkm2* ^+/+^ mice. N = 5 animals per group; Mean ± SEM; *p < 0.05; ***p < 0.005.

With the understanding that PKM2 is modulated during experimental retinal detachment (Fig. 5), we proceeded to explore the effect photoreceptor-specific, *Pkm2* conditional knockout mice would have on photoreceptor survival during experimental retinal detachment. In rodent eyes, photoreceptor TUNEL staining peaks 3 days after experimental retina/RPE separation and correlates with long-term survival^23^. Therefore, mouse retinas were detached and harvested after 3 days. Interestingly, as each subsequent *Pkm2* allele was deleted, the amount of TUNEL-positive cells in the ONL decreased. Retinal detachment in *Pkm2* ^−/−^ mice resulted in an approximately 85% decrease in the amount of TUNEL-positive cells in the ONL as compared to *Pkm2* ^+/+^ mice, which was statistically significant (Fig. 6a,b). We next sought to determine if the decrease in TUNEL staining corresponded with a decrease in caspase 8 activation, which has been previously validated as a marker of the extent of detachment-induced photoreceptor cell death^24^. At 3 days after experimental retinal detachment, caspase 8 activity was statistically significantly less in the *Pkm2* ^−/−^ mice as compared to *Pkm2* ^+/+^ mice (Fig. 6c).

To determine if this decrease in TUNEL staining and caspase activation corresponded with long-term photoreceptor survival, experimental retinal detachments were induced and eyes were harvested after 2 months (Fig. 6d). Photoreceptor cell counts were assessed by the number of photoreceptor nuclei in the ONL and normalized to the total retinal area. There was a significant decline in the number of photoreceptors in the detached retinas at 2 months compared to the attached retina controls for both the *Pkm2* ^+/+^ and *Pkm2* ^−/−^ mice (Fig. 6e). However, photoreceptor survival was significantly greater in detached retinas in *Pkm2* ^−/−^ mice as compared to *Pkm2* ^+/+^ mice (Fig. 6e). After 2 months, a 37% decline in photoreceptor survival was observed in the detached retinas as compared to attached retinas in *Pkm2* ^+/+^ mice while a 20% decline in photoreceptor survival was observed in *Pkm2* ^−/−^ mice. *Pkm2* ^−/−^ mice therefore showed 46% less photoreceptor cell death in experimental retinal detachment as compared to *Pkm2* ^+/+^ mice. Similar results were obtained for ONL area (Fig. 6f), further demonstrating that the *Pkm2* ^−/−^ mice not only reduce entrance into the apoptotic cascade (Fig. 6b,c) but also improve long-term photoreceptor cell survival.

## Discussion

In this study, we show that a photoreceptor-specific, *Pkm2* conditional knockout mouse results in an upregulation of *Pkm1* expression in the ONL as well as an increase in the expression of genes involved in glucose metabolism. These molecular changes produce small degenerative changes in the outer retina over time under baseline conditions as witnessed by the decrease in the scotopic a-wave amplitudes in the *Pkm2* ^−/−^ mice and concurrent changes in the outer retinal thickness measurements and cell counts. In contrast, under acute outer retinal stress, as produced by experimental retinal detachment, this same mouse model and its corresponding molecular changes showed reduced entrance into the apoptosis and/or necroptosis cascades and improved long-term photoreceptor survival. Since we showed that the phosphorylation status of PKM2 in rodent retina decreases after experimental retinal detachment to increase catabolic activity, the metabolic reprogramming observed in the retina of the *Pkm2* ^−/−^ mice may mimic this activation of PKM2 by substituting constitutively active PKM1 to circumvent acute apoptotic stress and ultimately, improve photoreceptor survival.

The *Pkm2* ^−/−^ mice selectively delete *Pkm2* from photoreceptors while still allowing *Pkm1* splicing and expression by crossing mice with Lox-P sites flanking PKM2-specific exon 10 (*Pkm2* ^*flox/flox*^) with Rho-Cre mice, in which Cre-combinase expresses specifically in rod photoreceptors^16,25^. In accordance with the rod-specific deletion of *Pkm2*, a decrease in the scotopic a-wave amplitude of the *Pkm2* ^−/−^ mice was observed (Fig. 4b), and this functional change coincided with small but significant anatomical changes in the outer retina of these animals (Fig. 3b,e and f). These small, chronic degenerative changes were most likely the result of slow cell death processes that could not be detected with TUNEL staining (Fig. 3g). A recent study by Chinchore *et al*. that briefly described the immunohistochemical findings in a similar mouse model supports these chronic outer retinal degenerative changes as it showed the nuclei in the ONL region to be disorganized with loss of typical columnar arrangement possibly due to cell death^15^. Additionally, Chinchore *et al*. report a slightly reduced rod OS length in this mouse model^15^. Taken together, our observations and data from others demonstrate that PKM2 function is important for photoreceptor survival as well as outer segment maintenance under baseline conditions.

Previous studies have shown that the mammalian retinal function is dependent on glycolysis^26^. Energy metabolism in the retina is dominated by aerobic glycolysis with greater than 80% of glucose consumed being converted into lactate^27,28^. Furthermore, aerobic glycolysis occurs primarily in photoreceptors^29^. Hence, it is not surprising that photoreceptors depend on glucose for survival and anabolic building blocks^30^. The photoreceptor-specific, *Pkm2* conditional knockout mouse model investigated in this report does not eliminate glycolysis. Rather, it alters a key regulatory node in the glycolytic pathway by removing the dynamically regulated PKM2 and upregulating its constitutively active isoform PKM1 (Fig. 1)^10,11^. Consequently, the abundance of glucose present in the outer retina under baseline conditions may have allowed the photoreceptors to meet the majority of their metabolic demands without causing an overt outer retinal phenotype. Likewise, we cannot exclude that other energy substrates, such as fatty acids, fueled the photoreceptors to meet metabolic demands and minimize degenerative changes^31^. Finally, altered gene expression in the photoreceptors may have helped redirect glycolytic intermediates into biosynthetic pathways in the absence of the PKM2, which is key to the promotion of anabolism in photoreceptors, circumventing drastic degenerative changes in the outer retina^15^. To this end, we observed upregulation of many genes involved in glucose metabolism in the *Pkm2* ^−/−^ mice (Fig. 2b). Specifically, genes involved in the non-oxidative portion of the pentose phosphate pathway were upregulated (Table 1), which may allow for shuttling of glycolytic intermediates into biosynthetic pathways that produce nucleotides or certain amino acids. Additionally, genes involved in the citrate shuttle (*Acly*, *Mdh1*, and *Mdh2*) that plays a role in moving acetyl-CoA from the mitochondria to the cytosol for fatty acid synthesis were upregulated (Table 1).

While *Pkm2* ^−/−^ mice showed small, chronic degenerative changes under baseline conditions, when exposed to acute outer retinal stress in the form of experimental retinal detachment^21,22^, photoreceptor survival was increased as compared to *Pkm2* ^+/+^ mice (Fig. 6). As determined in this study, *Pkm2* ^−/−^ mice upregulate *Pkm1* expression in the ONL, have greater pyruvate kinase activity, and increase the expression of genes involved in glucose metabolism (Figs 1, 2b). PKM1 has constitutively high activity while allosteric and post-translational regulatory events allow PKM2 to switch between a high- and low-activity state^10,11,32^. In the absence of PKM2, which is a key regulatory node in aerobic glycolysis, the constitutively high activity and lack of allosteric regulation of PKM1 coupled with the increased expression of genes involved in glucose metabolism, specifically those of glycolysis and the TCA cycle (Table 1), suggests a metabolic state that is associated with increased glycolytic flux, ATP synthesis, and catabolic metabolism^10^. Similarly, we showed that experimental retinal detachment in rats results in a decrease in the phosphorylation status (Y105) of PKM2 (Fig. 5). It has been previously shown that increased phosphorylation of PKM2 (Y105) decreases catalytic activity in the retina, and Y105 phosphorylation disrupts the formation of the active tetrameric PKM2^13,20^. In contrast, inhibition of FGF receptor signaling, which has been shown to regulate phosphorylation of PKM2 at Y105, or replacement of this tyrosine residue with a phenylalanine to abolish phosphorylation increases the catalytic activity of PKM2^15,20^. Furthermore, PKM1 and activated PKM2 have the same quarternary structure and similar kinetic parameters^33,34^. Taken together, a decrease in phosphorylation of PKM2, as seen during experimental retinal detachment, would therefore increase catalytic activity, which is associated with ATP synthesis and catabolic metabolism. Additionally, genes involved in glucose metabolism (*Hk2*, *Pfk*, and *Suclg2*) have been previously shown to have significantly different transcript levels after retinal detachment in rats^35^. As such, the metabolic reprogramming that occurred in the *Pkm2* ^−/−^ mice essentially recapitulated what the normal retina was attempting to achieve during acute outer retinal stress, which is to optimize ATP production to stave off cell death. In the normal retina, dephosphorylating a critical mass of PKM2 in order to shift metabolism from anabolic to catabolic requires time after metabolic insult. However, the *Pkm2* ^−/−^ mice did not have this time delay since the metabolic changes were already present under baseline conditions prior to the experimental detachment (Figs 1, 2b). As a result, the retina and specifically, the photoreceptors, of the *Pkm2* ^−/−^ mice may have been primed to better combat early apoptotic stressors leading to improved survival. As such, caspase 8 activity was decreased in the detached retinas of *Pkm2* ^−/−^ mice as compared to *Pkm2* ^+/+^ mice after 3 days (Fig. 6c). However, considering TUNEL staining detects DNA fragmentation in apoptosis and/or necrosis, alteration of other death pathways, such as the caspase independent pathway induced by apoptosis-inducing factor (AIF) or receptor-interacting protein (RIP) kinase-dependent necrosis pathways, cannot be excluded^19,36^.

In summary, we show that metabolic reprogramming in a photoreceptor-specific, conditional PKM2 mouse model results in small, chronic degenerative outer retinal changes under baseline conditions while providing improved photoreceptor survival during acute outer retinal stress. Mutations in enzymes important to energy metabolism and purine metabolism have been linked to retinal degenerations^37^. Considering photoreceptors have little reserve capacity to generate ATP and as a result, are susceptible to small changes in energy homeostasis, disruption of nutrient availability and metabolic regulation may be a unifying mechanism in photoreceptor cell death^37–39^. To that end, recent studies have demonstrated that reprogramming metabolism and enhancing glycolysis may prove to be an inventive therapeutic strategy for photoreceptor neuroprotection in retinal degenerations^8,40^. Our study gives credence to this strategy and shows it may be applicable to a multitude of retinal disorders, such as retinal detachment, where photoreceptor cell death is the ultimate cause of visual loss.

## Materials and Methods

### Materials

#### Antibodies

β-Actin (A5316-100UL, Sigma-Aldrich Co. LLC), PKM2 (Cat.#4053S, Cell Signaling Technology, Danvers, MA), pPKM2 (Y105) (Cat.#3827S, Cell Signaling Technology, Danvers, MA), PKM1 (Cat.#7067S, Cell Signaling Technology, Danvers, MA), PKM1/2 (Cat.#3186, Cell Signaling Technology, Danvers, MA). Secondary antibodies were anti-rabbit, anti-mouse, or anti-goat IgG-HRP.

#### Animals

All animals were treated in accordance with the Association for Research in Vision and Ophthalmology (ARVO) Statement for the Use of Animals in Ophthalmic and Vision Research. The protocol was approved by the University Committee on Use and Care of Animals of the University of Michigan (Protocol number: PRO00005497). To study the role of PKM2 in photoreceptor survival, *Pkm2* was selectively deleted from photoreceptors while still allowing *Pkm1* splicing and expression by crossing mice with Lox-P sites flanking *Pkm2*-specific exon 10 (*Pkm2* ^*flox/flox*^, Jackson Laboratories, Bar Harbor, ME) with Rho-Cre mice (courtesy of David Zacks, MD, PhD), in which Cre-combinase expresses specifically in rod photoreceptors. *Pkm2* ^*fl/fl*^ and Rho-Cre mice have been previously described^16,25^. These mice are on a C57BL/6 J genetic background. To ensure the phenotype is due to the loss of the gene of interest, these mice were screened for and lack the *rd8* mutation. Brown-Norway, adult rats were also utilized for experimental models of retinal detachment. All animals were housed at room temperature with 12-hour light and 12-hour dark cycle.

#### Chemicals

All reagents were analytical grade and purchased from Sigma (St. Louis, MO).

### Functional Assessment Testing

#### Optokinetic Tracking

A virtual-reality optokinetic tracking system (OptoMotry, CerebralMechanics, Inc., Alberta, Canada) was used to test visual acuity. Mice were placed on a pedestal inside a chamber consisting of four computer monitors, and were allowed to move freely. An alternating rotating sine wave grating stimulus was presented on the screens, in 3D space. The mice tracked the stimulus reflexively until it was no longer visible to them. This tracking was one-directional, temporal to nasal, so the acuity of both eyes was tested. The grating was fixed at the eyes of the mouse by constantly re-centering the virtual cylinder on the head. The tester recorded the presence and absence of tracking, and a simple staircase method was used to determine the highest level of spatial frequency (“acuity”) visible to the mice. The tests were done at 100% contrast with a drift speed of 12 deg/sec, starting at a spatial frequency of 0.042 cyc/deg.

#### Optical Coherence Tomography

A spectral domain ophthalmic imaging system (SD-OCT, Bioptigen, Morrisville, NC) was used to non-lethally measure retinal thickness in each mouse model. The mice received 0.5% tropicamide drops to stimulate eye dilation. The mice were anesthetized using an intramuscular injection of ketamine (50 mg/kg bodyweight) and xylazine (5 mg/kg bodyweight). One rectangular scan was done on each eye, of each animal. The scans were processed using the Bioptigen Diver software. Outer retinal thickness measurements (inner-most aspect of outer plexiform layer to inner aspect of retinal pigment epithelium) and outer segment equivalent length measurements (OSEL, inner segment/outer segment junction to retinal pigment epithelium inner surface) were obtained at four points, 0.35 mm from the optic nerve head according to the template in the Diver software. The average of the 4 values was used for analysis.

#### Electroretinography

A Diagnosys Espion E^2^ Electrophysiology System (Diagnosys, Lowell, MA) was used to assess retinal function. The mice received a drop of 0.5% tropicamide to stimulate eye dilation and a drop of 0.5% proparacaine to numb the eyes. The mice were anesthetized using an intraperitoneal injection of ketamine (50 mg/kg bodyweight) and xylazine (5 mg/kg bodyweight). The electroretinogram (ERG) was recorded using a small contact lens that is lightly placed on the surface of the cornea; it was cushioned with a drop of 2.5% Goniosol. A scotopic protocol was conducted using the stimuli I16. A 10-minute light adaption period preceded the photopic protocol. The photopic protocol consisted of the I16 intensity. During testing, body temperature was maintained at 37–38 degrees Celsius by a heating element.

### Experimental Model of Retinal Detachment

Detachments were created in Brown-Norway, adult rats as well as in the photoreceptor-specific, conditional knockout mice as previously described^21,22,41^. Briefly, rodents were anesthetized with a mix of ketamine (100 mg/mL) and xylazine (20 mg/mL), and pupils were dilated with topical phenylephrine (2.5%) and tropicamide (1%). A 25-gauge needle was used to create a sclerotomy located 1–2 mm posterior to the limbus with care taken to avoid lens damage. A subretinal injector was introduced through the sclerotomy into the vitreous cavity and then through a peripheral retinotomy into the subretinal space. Sodium hyaluronate (10 mg/mL) (Abbott Medical Optics, Healon OVD) was slowly injected to detach the neurosensory retina from the underlying retinal pigment epithelium (RPE). In all experiments, approximately one-third to one-half of the neurosensory retina was detached. Detachments were created in the left eye. The right eye served as the control, with all the steps of the procedure performed, except for introduction of the subretinal injector and injection of the sodium hyaluronate.

### TUNEL Staining and Histology

Mice were euthanatized and the eyes were enucleated. Whole eyes were fixed overnight at 4 °C in phosphate-buffered saline with 4% paraformaldehyde (pH 7.4). The specimens were embedded in paraffin and were then placed in a tissue processor (Tissue-Tek II; Sakura, Tokyo, Japan) for standard paraffin embedding. Eyes were then sectioned at a width of 6 μm on a standard paraffin microtome (Shandon AS325, Thermo Scientific, Cheshire, England). TUNEL staining was performed on the sections using DeadEnd™ Colorimetric TUNEL System (Cat.# G7360, Promega Corporation Madison, WI, USA) according to the manufacturer’s instructions. TUNEL-positive cells in the outer nuclear layer were counted in a masked fashion. For outer nuclear layer (ONL) cell count and retinal area measurements, paraffin sections were stained with 0.5% toluidine blue in 0.1% borate buffer.

### Caspase Activity Assay

Attached and detached retinas were harvested after 3 days and homogenized using a sonicator at 20% power for 10 pulses in lysis buffer (20 mM MOPS, pH 7.0; 2 mM EGTA; 5 mM EDTA; 0.1% Triton X-100). 1 tablet of protease inhibitor (Complete Mini; Roche Diagnostics, Indianapolis, IN) and 1 tablet of phosphatase inhibitor (PhosSTOP; Roche Diagnostics, Indianapolis, IN) per 10 mL were added to the lysis buffer. The homogenates were centrifuged at 10,000 *g* for 10 minutes at 4 °C, and total protein concentration of the supernatant was determined using a Micro BCA protein assay kit (ThermoScientific, Rockford, IL). A luminescent assay kit (Caspase 8 Glo Assay, Promega, Madison, WI) was utilized to measure caspase 8 activity according to the manufacturer’s instructions. In short, 50 μg of protein was incubated with substrate in a white-walled 96 well-plate at room temperature for 1 hour. The attached retinas served as controls. Luminescence was measured in a plate reader luminometer (Turner Biosystems, Sunnyvale, CA).

### Cell Counts and Retinal Area Measurements

Retinal images were obtained using Leica DM6000 microscope (Leica Microsystems Wetzlar, Germany). For toluidine blue–stained specimens through the plane of the optic nerve, the total number of cells in the ONL were measured using a macro program in ImageJ software. The total area of the ONL and retina (from the outer edge of the ONL to the inner limiting membrane) was measured using ImageJ in high-power field (40×) images. Photoreceptor inner and outer segments were not included in the total retinal area measurement, given the variable retraction or stretch of these elements upon processing, which does not correlate with viability of the photoreceptors. Using this macro program, the entire section is counted or the area determined from one end of the retina to the other. Normalization of ONL cell count or ONL area to the total retinal area of each section was performed to account for possible differences in angles of sectioning and to allow for inter-sample comparison. Data are represented as mean +/− SEM.

### Immunohistochemistry

Immunohistochemistry was performed on sections obtained from paraffin-embedded retinas using standard protocol. Epitope unmasking was accomplished by incubating the sections in boiling sodium citrate Antigen Retrieval buffer. The primary antibody for PKM2 was at a concentration of 10 µg/ml in 1% normal goat serum, 1% BSA in PBST. The primary antibody for PKM1 was at a concentration of 2.5 µg/ml in 1% normal goat serum, 1% BSA in PBS-0.1% Trition X-100. Secondary antibody concentration was 1:1000 in 3% BSA in PBS.

### Western Blot Analysis

Retinas from experimental rodent eyes were dissected from the RPE-choroid, homogenized, and lysed in RIPA Lysis and Extraction Buffer (Catalog number: 89900, Life Technologies Corporation, Grand Island, NY). 1 tablet of protease inhibitor (Complete Mini; Roche Diagnostics, Indianapolis, IN) and 1 tablet of phosphatase inhibitor (PhosSTOP; Roche Diagnostics, Indianapolis, IN) per 10 mL were added to the lysis buffer before use to prevent proteolysis and maintain protein phosphorylation. The cellular debris was removed by low speed centrifugation and protein concentrations of supernatants were determined by Pierce BCA Protein Assay Kit (Life Technologies Corporation, Grand Island, NY). Protein samples were separated by SDS NuPAGE Novex 10% gels (Invitrogen, Carsbad, CA, transferred onto Polyvinylidene difluoride membranes that were blocked in blocking buffer (5% nonfat dry milk in phosphate- buffered saline and 0.1% Tween 20) for 1 hr, incubated with primary antibody, washed, and incubated with horse radish peroxidase-conjugated secondary antibody, developed with SuperSignal™ West Dura Extended Duration Substrate, and image captured digitally on Azure c500 (Azure biosystems Dublin, CA). Densitometry measurements were performed using Image J software.

### Pyruvate Kinase Activity Enzyme Assay

A continuous, enzyme-coupled assay, which uses lactate dehydrogenase (LDH) and measures the depletion of NADH via absorbance at 340 nm was utilized to determine the pyruvate kinase activity in mouse retinal lysates. Retinas from experimental mouse eyes were dissected from the RPE-choroid, homogenized, and lysed in RIPA Lysis and Extraction Buffer (Catalog number: 89900, Life Technologies Corporation, Grand Island, NY). The assay was carried out using 5 microliters of mouse retinal lysate in an enzyme buffer mixture (50 mM Tris-HCl, pH 7.4, 100 mM KCl, 5 mM MgCl_2_, 1 mM ADP, 0.5 mM PEP, and 0.2 mM NADH) and 8 units of LDH similar to what has been previously described^13^. Pyruvate kinase activity was normalized to total PKM protein levels.

### Quantitative Real-Time PCR

Total RNA was purified from retinal tissues of mice using RNeasy Mini kit (Qiagen, Cat No./ID: 74104) and QIAshredder (Qiagen, Cat No./ID: 79654). cDNA was synthesized using RT^2^ First Strand Kit (Cat. No./ID: 330401) according to the manufacturer’s instructions. Quantitative PCR was performed in at least triplicate for the different experimental groups using the Mouse Glucose Metabolism RT^2^ Profiler™ PCR Array (Qiagen, Cat No./ID: PAMM-006Z). This PCR array allows simultaneous profiling of 84 genes involved in glucose metabolism and includes housekeeping genes for normalization. Reactions were performed and monitored using a CFX96 real time PCR system (Bio-Rad Laboratories), 1 cycle at 95 °C for 10 min followed by 40 cycles at 95 °C for 15 sec, 60 °C for 1 min. Data analysis was performed using the RT^2^ Profiler™ PCR Array Data Analysis Template from Qiagen. Those genes whose relative expression level was low or not detected in all experimental groups were excluded from analysis. Data were normalized to the housekeeping genes *Actb* and *B2m*.

### Data Analysis

Results are expressed as mean ± SEM. Data was analyzed using Student *t* test or one-way ANOVA followed by Bonferroni post hoc test. A value of *P* < 0.05 was considered significant. Prism 6.0 (GraphPad Software, San Diego, CA) was used for all statistical analysis.

### Data Availability

The datasets generated during and/or analysed during the current study are available from the corresponding author on reasonable request.
